# Innate and Adaptive Immune Responses in HELLP Syndrome

**DOI:** 10.3389/fimmu.2020.00667

**Published:** 2020-04-15

**Authors:** Violeta Stojanovska, Ana Claudia Zenclussen

**Affiliations:** Experimental Obstetrics and Gynecology, Medical Faculty, Otto von Guericke University Magdeburg, Magdeburg, Germany

**Keywords:** HELLP, innate immunity, adaptive immunity, complement system, pregnancy

## Abstract

Innate and adaptive immune involvement in hemolysis, elevated liver enzymes and low platelet (HELLP) syndrome is an understudied field, although it is of high clinical importance. This syndrome implies a risk of serious morbidity and mortality to both the mother and the fetus during pregnancy. It was proposed that HELLP syndrome occurs in a circulatory inflammatory milieu, that might in turn participate in a complex interplay between the secreted inflammatory immunomodulators and immune cell surface receptors. Meanwhile, reported immune cell attenuation during HELLP may consequently lead to a prolonged immunoactivation and tissue damage. In this regard, learning more about the immune components of this syndrome should widen the understanding of the HELLP pathophysiology and eventually enable development of novel immune-based therapeutics. This review aims to summarize and discuss the recent and previous findings of the innate and adaptive immune responses during HELLP in order to update the current knowledge of the immune involvement in HELLP pathogenesis.

## Introduction

The maternal immune system has an essential role in pregnancy maintenance and it is furthermore in control of pregnancy development, progression and outcome (1). Pregnancy is a unique physiological state which results in maternal immune tolerance of the developing semi-allogeneic fetus and maternal immune activation in order to induce metabolic adaptation of the mother to meet the increased nutrient demands of the fetus (2). Moreover, a mild systemic inflammatory response is detected in normal pregnancy (3). Tightly controlled interactions between the immune, endocrine, and metabolic system during pregnancy are necessary in order to establish proper placentation, nurture and immune homeostasis (4, 5). Disbalance in these interactions is considered to be a base of many pregnancy-associated disorders including **H**emolysis, **E**levated **L**iver enzymes, **L**ow **P**latelet (HELLP) syndrome.

Hemolysis, elevated liver enzymes, low platelet is a life-threatening, rapidly progressive pregnancy-associated disorder that usually occurs in 1–6 women per 1000 pregnancies (6). The clinical symptoms are non-specific and challenging for accurate and fast diagnosis. Two major classification systems (established in the ‘80s) based on laboratory results are used to categorize the patients. In the Tennessee classification, concentrations of lactate dehydrogenase (LDH), aspartate- and alanine-aminotransferase (AST, ALT) and platelet counts are used to assess the degree of hemolysis, liver damage and thrombocytopenia, respectively. With the Mississippi Triple Class System further categorization of the disease is done based on the severity of thrombocytopenia (7, 8). Although traditionally HELLP syndrome was considered to be a severe form of preeclampsia (a disorder characterized by development of hypertension and proteinuria in pregnancy), several research lines are counteracting to this notion mainly because HELLP is associated with hypertension and/or proteinuria only in 80% of the cases and shows different cytokine activation and aggravated placental vascular lesions (9–15). Furthermore, the etiopathogenesis of this syndrome is complex and still not completely understood thus, additional and more specific/sensitive laboratory criteria are needed for up-to-date HELLP screening, diagnosis and treatment.

As mentioned, the HELLP syndrome can share some pathophysiological traits with preeclampsia (16). The development of preeclampsia is attributed by deficient spiral artery remodeling and shallow trophoblast invasion. When spiral arteries fail to remodel, either due to inefficient trophoblast invasion and/or inadequate trophoblast induced endothelial cell apoptosis (17), which is primed by immune cells (18), will lead to placental ischemia. This, in turn, is accompanied by an increased release of antiangiogenic factors and activated endothelium that in turn will lead to development of hypertension and proteinuria. In some cases, progress to multiorgan microvascular injury and liver dysfunction then occurs (16, 19). In patients with the HELLP syndrome, the fetus can also contribute to initiation of the disease, through abnormal oxidation of fatty acids and transfer of the subsequent metabolic intermediate into the maternal circulation leading to liver and vascular malfunction (20). Interestingly, these changes occur only when the fetus has an inherited metabolic defect in mitochondrial fatty acid oxidation, which is not the case for all HELLP pregnancies. Moreover, HELLP syndrome is associated with leukocytosis (21), and an excessive inflammatory response (22, 23) with increased concentrations of proinflammatory and decreased concentrations of anti-inflammatory cytokines (24, 25). The fact that corticosteroid administration can halt the progression of the disease (26), suggest that immune system might be involved in the etiopathogenesis of HELLP. In the present review, we aim to compare how the immune system operates during uncomplicated pregnancies and in pregnancies complicated by the HELLP syndrome as reported in the literature. Moreover, we will discuss the detailed aspects of the innate and adaptive immunity by which aberrant activation may predispose the host to hematological and hepatic complications of pregnancy, as seen in HELLP syndrome. In the end, we focus on the currently available experimental models and the possibility for immune-related HELLP research on them.

## Innate Immune Component in HELLP

The innate immune system is the first line of defense against pathogens and it is comprised of cellular and molecular mechanisms that always act in a similar way against infection. All cellular components of the innate immune system have the ability to recognize microbial or damage-associated molecular patterns (known as PAMPs and DAMPs) via pattern recognition receptors (PRRs) or via specific proteins such as the complement system (27). Knowing that in HELLP syndrome several organs are affected and can serve as a source of DAMPs (28, 29), it is interesting to know to what extent each of the innate immune components are involved in its etiopathogenesis.

### Neutrophils Are Mediators of Liver and Endothelial Damage in HELLP

Neutrophils are short-lived polymorphonuclear leukocytes that act as effector cells of the innate immunity via phagocytosis, release of cytotoxic enzymes and neutrophil extracellular traps (NETs) and recruitment of other effector cells (30). In addition, neutrophils play a major role in tissue damage and repair (31) by promotion of excessive inflammatory response.

Liver damage is one of the hallmark signs of HELLP syndrome, however, it is still unclear why and how it occurs. The most studied initiator for liver damage so far is the ischemic-reperfusion injury. As a consequence of this, hepatocytes show apoptotic and necrotic changes, just as reported in the HELLP syndrome (32, 33). As a result of the injury, neutrophil infiltration in the sinusoids and post-sinusoidal venules occurs with concomitant extravasation in the liver parenchyma (34). However, there is a limited evidence about the involvement of neutrophils mediated liver damage in HELLP. In a study from Halim et al. it was reported that liver tissue from HELLP patients was infiltrated with neutrophils in comparison to controls and liver tissue samples from acute fatty liver syndrome patients (35). Another study reported that neutrophils-to-lymphocyte ratio was higher in patients with HELLP syndrome majorly due to increased neutrophils in peripheral blood samples (13). This implicates that in HELLP patients, neutrophil counts are likely increased in the liver and in the peripheral circulation. However, another study reported that neutrophils isolated from peripheral blood from HELLP patients did not show increased reactive oxygen species (ROS) production compared to controls. On the contrary, these neutrophils had diminished ROS production (36), meaning that either these cells show aberrant functionality or were completely exhausted during the course of HELLP. In addition, incubation of neutrophils with sera from HELLP patients show increased ROS production, suggesting that certain factor or factors present in the serum can lead to an increased neutrophil activation (36).

Endothelial activation occurs in HELLP and increased active von Willebrand factor (vWF) levels can contribute to thrombocytopenia in the HELLP syndrome (37). Hulstein et al. hypothesized that the presence of placental debris in the circulation may lead to this activation, which was later proven by Shen et al. that trophoblast debris derived from preeclamptic placentas induced endothelial cell activation (38). Moreover, microparticles derived from placental tissue are able to efficiently activate neutrophils *in vitro* to generate NETs (39). However, it is not known whether neutrophils infiltration can also lead to endothelial activation in HELLP. Recently, it was reported that NETs can promote endothelial cell activation via IL-1a and cathepsin G that in turn will lead to an increased thrombogenicity (40), implying that neutrophils might mediate the prothrombotic effect of endothelial activation as registered in HELLP patients.

### Monocytes and Macrophages in HELLP

Monocytes are short-lived leukocytes that elicit immune responses via phagocytosis, antigen-presentation and cytokine production (41, 42). When recruited to a certain tissue, they are capable to differentiate into macrophages or dendritic cells. Macrophages, as terminally differentiated monocytes, are able to induce immune responses in the same way as the monocytes, plus have an additional ability of self-renewal as observed in Hofbauer cells in the placenta and Kupffer cells in the liver (43). In uncomplicated pregnancies, monocyte counts are increased toward the end of the pregnancy and they show functional changes (44), such as increased production of ROS and decreased phagocytic activity and cytokine production (45–47). As the pregnancy progresses, the number of Hofbauer cells changes, showing a peak at the second trimester and gradually declining toward the third trimester (48).

It was reported that during HELLP syndrome the monocyte population is decreased (49) and Hofbauer cells were significantly increased in placentas from HELLP patients, detecting most of the macrophages nearby the vascular area of the villus stroma (50). These opposing findings between normal and HELLP pregnancies suggest that monocytes and macrophages are affected during HELLP syndrome. Moreover, monocytes have the ability to ingest damaged erythrocytes, and via chemotactic signaling they can get accumulated in the liver and be transformed into macrophages responsible for iron turnover (51). Knowing that erythrocyte destruction is increased in HELLP, it would be interesting to know whether this contributes to increased monocyte activation and macrophage overpopulation in the liver. Interestingly, another study confirmed that liver macrophages are responsible for liver damage in an experimental model of HELLP obtained by low dose administration of lipopolysaccharide (52). Treatment with selective inhibitor of macrophages was indeed successful in omitting the symptoms in this experimental model of HELLP (52).

### Dendritic Cells in HELLP

As antigen-presenting cells, dendritic cells (DC) are part of the innate immune system and are able to induce primary immune responses or tolerance (53) by conveying the information toward the adaptive immune system. The dendritic cells can be divided into two subgroups; DC-1 or myeloid dendritic cells which are the largest population in the peripheral blood system and DC-2 cells which are lymphoid and can lead the differentiation of T cells into Th2 cells (54, 55). In early pregnancy, the number of DC-1 in peripheral circulation is low, but increases as the pregnancy progresses (55), forming up to 70% of the total circulating dendritic population (56). Moreover, a shift in dendritic populations can be observed in the presence of different types of cytokines such as IL-4 and TNF-alpha (55). Locally in the placenta, DC are scattered throughout the placental bed in relatively low numbers displaying low proliferative capacities (57), indicating that in the placenta, mostly immature, thus tolerogenic, DC are present. Although the role of DC in feto-maternal tolerance is still unclear, several lines of research propose that DC modulate the maternal immunity toward Th2 type responses in order to maintain the immune tolerance (58, 59). Unfortunately, there are not many studies evaluating the number or the functionality of DC in HELLP syndrome. Scholz et al. reported an upregulation and downregulation of certain DC markers in paraffin-embedded placental tissue from HELLP patients (60), whereas these differences were not observed in uncomplicated pregnancies and neither in pre-eclamptic samples. Since, platelet count and functionality are changed during HELLP syndrome (6, 61) and are also involved in proper DC differentiation and activation (62, 63), it is important to further evaluate to what extent DC play a role in the immunomodulatory mechanisms of HELLP.

### Complement System Involvement in HELLP

The complement system as part of the innate immune system is comprised of cell bound and free proteins that can interact in a cascade of activation. Complement activation can occur via three pathways depending on the trigger factors including; classical, lectin and alternative pathway (64), resulting in inflammation, cell death or facilitated phagocytosis with consequent clearance of cell debris and pathogens (65). Most of the complement proteins are produced by the hepatocytes (66), however, extrahepatic production was detected as well (67, 68). This, in turn, might provide necessary protection against infections in vulnerable tissues. Moreover, the complement system interacts with the coagulation system and can induce platelet activation via C3a and C5a components (69).

Throughout the pregnancy, the complement system does not only protect the organism against infections, but it also orchestrates the optimal placental development (64). In the last third of uncomplicated gestations, several complement components and activation products show higher concentrations in comparison to non-pregnant controls and increased complement activation via the classical and lectin pathway (70). Moreover, several complement components, among which C1q, C3, and C4, are locally produced by cytotrophoblasts and decidual cells (67, 71), promoting local defense mechanisms and controlled trophoblast invasion of the placenta. Furthermore, complement deficiencies can lead to aberrant placentation and recurrent pregnancy loss (72, 73). Recently, several lines of research attribute the HELLP syndrome as a complement-amplifying condition (CAC) (74, 75), reinforcing the role of the complement system in its pathogenesis.

Disturbances in the complement components and activity were identified in HELLP patients, although that was not the case in all studies. Screening of HELLP patients in several studies, showed either increased activation of the classical or alternative complement pathway or showed a deficiency of proteins that regulate the complement system (76–79). Moreover, the introduction of immunoglobulin therapy in a limited number of patients with eculizumab, a humanized monoclonal IgG antibody against the complement protein C5, leads to alleviation of the hematological, hepatic and vascular malfunctions that are present in HELLP syndrome (80, 81). On the contrary, genetic mutations in the alternative complement pathway, although initially proposed as important in mediating HELLP pathogenesis were detected only in a few patients with HELLP (77, 82). Another study focusing on the complement C3 component and regulatory protein complement factor H in serum from HELLP patients and control subjects showed no changes in the concentrations between the groups (83). Even though the involvement of the complement system in mediating HELLP syndrome is plausible, yet is not as crucial as previously suspected.

### Role of NK Cells in HELLP

Natural killer cells are a group of lymphocytes that have either cytotoxic or cytokine producing properties (84). They get activated based on ligand interaction with the surface activating or inhibitor receptors (85). These receptors are of particular importance in order to prevent the killing of healthy “self” cell. NK cells also produce a variety of chemokines and cytokines, such as; INFg, TNFa, IL-10 and IL-8 (86, 87). In the human endometrium and placental bed there is a special population of uterine NK cells (uNK) that are able to produce cytokines, but still are morphologically different than the ones present in peripheral blood, e.g., pbNK (88). This is mainly because they can secrete vascular endothelial growth factor C, placental growth factor (PlGF), angiopoietin 2 (ANG2) and cytokines involved in angiogenesis (89). They are abundant in the first trimester of pregnancy and their number decreases from mid-gestation onward (90). In spite of being part of the innate immune system it was suggested that these cells might act as trained memory cells (91). Besides, uNKs cannot kill.

So far it is known that dysregulation of either peripheral or uterine NK cells is associated with several reproductive conditions, including: infertility, recurrent pregnancy loss and preeclampsia (89, 92). To the best of our knowledge, there are no studies reporting or studying the effect of HELLP syndrome on the number or functionality of pbNK or uNK cells. NK cells may however emerge as important in the pathophysiology of HELLP, because they can mediate cell apoptosis via secretion of granzymes, and apoptosis is present in the histopathological data from livers of HELLP patients. Furthermore, platelets interact with NK cells, as known from recent oncological research, either via presenting molecules or antigens such as MHC-1 that can be recognized by the NK cells (93) or via platelet derived ectosomes (94). Knowing that platelets can impair/reduce NK cell reactivity either via ectosomes release and via TGF-b1 signaling, it appears important to understand whether NK cell function is affected when thrombocytes are depleted.

## Adaptive Immune Component in HELLP

The adaptive immune system represents the most specialized protection against pathogens and is also characterized by the generation of memory immune cells. The immune response is mediated via two types of responses: the cellular and the humoral response. The cellular one is mediated by T cells, whereas the humoral one is mediated via production of antibodies produced by the B cells (95). Patients with HELLP syndrome have an increased recurrence risk to develop HELLP syndrome in the next pregnancy as well, although it reappeared in less than 6% of the subsequent pregnancies (96, 97). This implies that the genetic implication of recurrence is subtle and might be dependent on immune system disturbances. Below, we will address what kind of incompetence of T and B cells are reported in the HELLP syndrome.

### T Cells in HELLP

T cells represent a large population of the adaptive immune response and depending on their surface molecules and mode of action, can be subdivided into cytotoxic T cells (CD8+) and helper T cells (CD4+). CD8+ T cells directly attack target cells and show immunosuppressive abilities by dampening the production of antibodies by B cells (98). Taken into consideration that CD8+ T cell proliferation increases by the end of pregnancy; they might be involved in the maintenance of fetal tolerance (99). The CD4+ T subset is known for its cytokine production and subsequent activation of macrophages and B cells. Based on their ability to produce different types of cytokines they can be subdivided into four subgroups; including Th1, Th2, Th17, and Treg cells (100). A delicate balance of Th1/Th2/Th17/Treg is necessary for uncomplicated pregnancy (100).

The pivotal role of Treg cells in developing tolerance in peripheral and transplantation tissues (101), suggested a role for Treg in establishing pregnancy tolerance, a fact that was first described in our laboratory (102). Thus, Treg are thought to prevent pregnancy complications where immune tolerance is affected such as in; infertility, miscarriage and preeclampsia (103–105). Moreover, animal studies showed that Treg depletion leads to an increased proinflammatory status in pregnancy (106) that hindered implantation, altered uterine artery function and increased fetal loss (102, 107). Knowing that most studies including samples from patients (105, 108–110), but not all (111, 112), report reduction in Treg cell expansion during preeclampsia, it is interesting to know whether this is also the case for the HELLP patients. In human studies, it was reported that Treg cell counts and suppressive activity was not affected in patients with HELLP syndrome (113, 114). However, the memory T cell differentiation was altered (115), proposing that the lower number of naïve Treg and reinforced differentiation into memory Treg in HELLP patients might preserve their immunosuppressive activity, however, this needs further evaluation.

Animal studies so far propose a more important role for T cells in HELLP pathogenesis. In an experimental HELLP model obtained by chronic infusion of antiangiogenic factors, it was shown that the overall concentration of CD4+ and CD8+ T cells is increased, while Th17 and Treg concentrations were comparable between the groups (116, 117). Later on, when these animals were treated with Abatacept, an antibody that blocks T cell activation, the biochemical parameters of HELLP syndrome were improved (118). However, the factor(s) leading to an increased T cell activation in HELLP, remains puzzling. It is interesting to consider that Fas-Fasl system is upregulated in HELLP syndrome (119–121) and one of its multiple roles besides promotion of apoptosis is also *per se* regulation of inflammatory response via activation and proliferation of CD4+ and CD8+ T cells (122, 123).

### B Cells in HELLP

B cells are known to modulate the immune responses by secretion of cytokines, autoantigen presentation and production of antibodies. In short, B cell population can be subclassified into B1, B2 and regulatory B (Breg) cells (124). Whereas B1 and B2 cell populations have the ability to produce polyreactive and adaptive antibodies, respectively, Breg population possess the ability to secrete IL-10 and IL-35 (125–128). However, a detailed characterization of B cell populations and functionality induced during pregnancy and pregnancy-associated disorders is still limited. Studies in mice revealed that B cell populations are dynamically changed in normal pregnancies by increasing mature B cells in the bone marrow and the spleen, and decreasing the numbers of pre/pro and immature B cells (125). Moreover, the immunoglobulin levels of IgM, IgA, and IgG in the peripheral circulation are also increased in murine pregnancy in comparison to non-pregnant mice (129). Studies in humans reported that absolute counts of B cell peripheral compartment in the last trimester of pregnancy and in term decidua are decreased in comparison to non-pregnant controls (130, 131).

HELLP is not typically described as an autoimmune disorder, however, it is highly prevalent in pregnant women with autoimmune diseases such as acute phospholipid syndrome, systemic lupus erythematosus and thrombotic thrombocytopenic purpura (132–135). To what degree antibodies can play a role in HELLP pathogenesis is still questionable. Previously, it was reported that cytotoxic and antiplatelet antibodies occur in the serum of HELLP patients (136). On the contrary, another study reported that in HELLP patients there are no platelet associated IgGs (137). Considering the wider spectrum of autoantibodies that might be present in pregnancy-associated disorders, Weitgasser et al. reported that 31% of the HELLP patients have different types of autoantibodies (antinuclear antibodies, anti-thyroid, etc.) in peripheral circulation in comparison to only 10% in control subjects (138). In animal studies, in an experimental model of HELLP syndrome obtained by anti-angiogenic disbalance it was reported that there was no difference in B cell counts in the circulation in comparison to a control group (116). However, there is a possibility of B cell subpopulations number variations and/or altered activation. Our lab reported that B-1a cells contribute to the production of autoantibodies, namely angiotensin II type 1 receptor autoantibodies AT1-AA (139), which were shown to be present in HELLP patients (26, 140, 141). This served as a background for the development of a novel experimental model of HELLP. When purified IgGs (containing AT1-AA) from HELLP patients and purified IgGs from control subjects were introduced to pregnant rats during mid-gestation, resulted in the development of the biochemical characteristics of HELLP (140). This sheds a light that AT1-AA are involved in the pathogenesis of HELLP, however to what extent and whether there is an interplay with B and T cell dysregulation needs further investigation.

## Current Experimental Models of HELLP

Animal models provide the distinctive opportunity to study the immune-related mechanistic traits of HELLP syndrome. Unfortunately, there is no uniform model so far that incorporates all the pathophysiological traits of HELLP. Moreover, most of the available ones were developed as models of preeclampsia (140, 142–144) that in the end turned to have exaggerated symptomatology, such as liver dysfunction and hematological abnormalities which are regularly absent in preeclamptic patients. This questions whether all models of preeclampsia in the end can serve as preclinical models of HELLP. In addition, only few have characterized the immunological traits in these models (52, 116).

The preeclamptic models so far that were evaluated for liver and hematological abnormalities (Table 1) include the following mechanisms of action; systemic inflammation (52), angiogenic disbalance (116), transfer of autoantibodies (140, 143), affected low oxygen sensing (144) and the combined model of angiogenic disbalance and impaired nitric oxide production (145). On the contrary, the model of reduced uterine perfusion pressure (RUPP) which is regularly used for preeclampsia research, does not show the biochemical characteristics of HELLP (146). Thus far, only the initial inflammation model of preeclampsia (147), was adjusted to mirror HELLP characteristics (52). Late gestation administration of 200 times higher LPS concentrations in rats resulted in laboratory and histological abnormalities, as registered in HELLP patients, including thrombocytopenia, hemolysis, elevated liver enzymes, hepatocellular necrosis, sinusoidal fibrin deposits and increased macrophage liver infiltration. Almost all symptomatology, except fetal loss, was reversed when the animals were pretreated with gadolinium III chloride which serves as selective macrophage inhibitor, proposing that indeed maternal immunological alterations can mediate HELLP pathogenesis (52). Arguably, although all animal models in one way or another depict the clinical characteristics of HELLP, still it remains uncertain whether the entire sequence of pathophysiological events is fully represented including the immunological disturbances as observed in HELLP patients.

**TABLE 1:** Overview of the current experimental animal models of hemolysis, elevated liver enzymes and low platelets (HELLP) syndrome.

**Mechanism of action**	**Strain**	**Method**	**HELLP traits**	**Immune responses**	**References**
Inflammation	SD rats	GD 17	↑AST, ALT and LDH	Macrophage infiltration in	(52)
		Systemic i.v. administration of 0.2 mg/kg LPS	↓platelets Liver necrosis, fibrin deposits	the liver	

Angiogenic	SD rats	GD 12-19	↑ALT, LDH	↑TNFa, IL-6, IL-17	(116)
disbalance		i.p. chronic administration of recombinant sFlt-1 and	↓platelets	↑ CD8+T cells in circulation and liver	
		sEng via osmotic pumps		↑ CD4+ T cells in circulation, liver and placenta	
				↓ Treg/Th17 ratio in circulation and liver	

Autoantibodies	SD rats	GD 10	↑ALT, LDH	Plasma ↑TNFa	(140)
transfer		i.v. IgG (containing AT1-AA) transfer	↓platelets Liver necrosis	Limited lymphocyte infiltration in the liver	

Low oxygen sensing disruption	C57Bl/6J mice	GD 8 Adenoviral overexpression of HIF-1a	↑AST, ALT, LDH ↓platelets Fibrin deposits in the liver	Lymphocyte infiltration in the liver	(144)

Angiogenic imbalance and impaired NO production	C57Bl/6J mice	Adenoviral overexpression of sFlt-1in non-pregnant endothelial NOS −/− mice These animals cannot maintain pregnancy	↑AST, ALT ↓platelet No changes in erythrocyte counts Liver necrosis and apoptosis, fibrin deposits	Neutrophil infiltration in the liver	(145)

*SD = Sprague Dawley; GD = Gestational day; AST = aspartate aminotransferase; ALT = alanine aminotransferase; LDH = lactate dehydrogenase; sFlt-1 = soluble Fms-like tyrosine kinase 1; sEng = soluble endoglin; AT1-AA = angiotensin II type 1 receptor autoantibodies; NO = nitric oxide; HIF = hypoxia inducible factor; i.v. = intravenous; i.p. = intraperitoneal.*

Treatment strategies for HELLP syndrome can also have a detrimental effect on the immune system and can be easily tested *in vivo*. However, this area is scarcely reported in the literature to date. For instance, in a meta-analysis of total of fifteen studies it was concluded that corticosteroids significantly prevent the platelet consumption and erythrocyte destruction (148) without tackling down the effect on the immune cells. It is a similar situation with the eculizumab treatment for HELLP syndrome. The study from Elabd et al. (81) shows that platelets, white blood cells counts and hepatological parameters are improved in HELLP patients, but detailed analysis of the effect on immune cells is missing. Knowing that potential treatments for HELLP syndrome are limited, identification of an appropriate one that will consider the whole pathophysiological feature is of major importance.

## Concluding Remarks and Future Prospects

**FIGURE 1:**
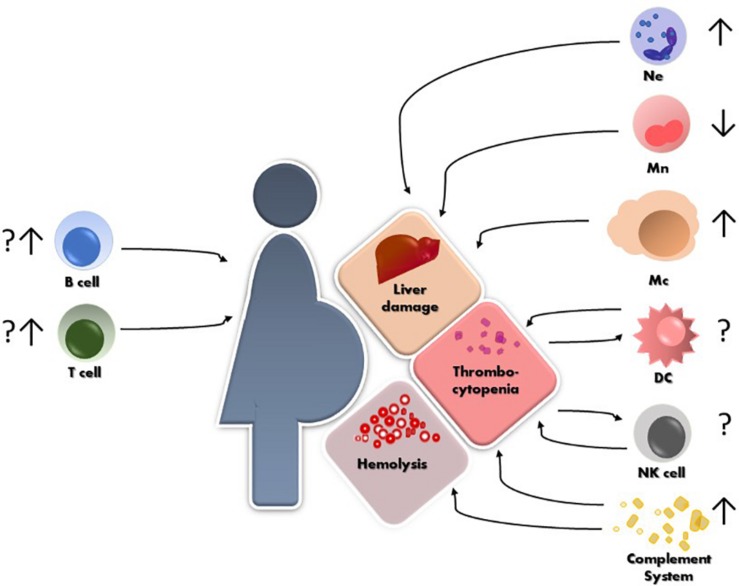
Schematic representation of HELLP syndrome and the reported alterations so far, of different immune cells and their possible contribution to the hepatic and hematological injuries. Ne = neutrophils, Mn = monocytes, Mc = macrophages, NK = natural killer cells, DC = dendritic cells.

The maternal immune system plays a critical role in several aspects of pregnancy, from defense against pathogens, through establishment of a suitable immune milieu for embryo implantation and placentation to specific tolerance of paternally derived antigens. We are just beginning to understand to what grade the immune system plays a role in the development of HELLP. What we have summarized in this review is that isolated reports cover all segments of the immune system as being in one way or another involved in the pathogenesis and the clinical course of HELLP (Figure 1). However, more conclusive data are necessary in order to unravel which factor precedes the other and what is the correct interplay that leads to the development of HELLP. Most importantly, working *in vitro*, *ex vivo* and *in vivo* models are necessary to tackle down the maternal or fetal component(s) that initiate the cascade of events leading to HELLP.

Taking into account that HELLP syndrome is a rare disorder and that all of the studies face the same limitation, such as having a small cohort studies, multicenter collaborations, a large multicentric cohort study (“big data”) might overcome this problem in future. Moreover, developing a specific translational experimental model of HELLP that can provide extensive inside into the immunomodulatory mechanisms underlining the syndrome is of vital importance. Proper identification of the immune disturbances and strategies to target the same, can ensure additional diagnostic and therapeutic perspectives for the HELLP syndrome.

## Author Contributions

VS wrote the manuscript and prepared the figure and table. AZ provided critical feedback.

## Conflict of Interest

The authors declare that the research was conducted in the absence of any commercial or financial relationships that could be construed as a potential conflict of interest.
